# Systematic In Silico Analysis of Interhelical Electrostatic Interactions in Leucine Zippers

**DOI:** 10.3390/molecules31173081

**Published:** 2026-09-01

**Authors:** MinSoung Kang, Suin Joung, Eunju Kwon

**Affiliations:** 1 Research Institute of Molecular Alchemy, Gyeongsang National University, Jinju 52828, Republic of Korea; mukbaque@gnu.ac.kr; 2 Applied Life Science, Gyeongsang National University, Jinju 52828, Republic of Korea; suin0412@gnu.ac.kr; 3 Division of Life Science, Gyeongsang National University, Jinju 52828, Republic of Korea

**Keywords:** leucine zipper, coiled coil, salt bridge, ionic repulsion, electrostatic interactions, protein engineering, interaction energy, in silico mutational scan

## Abstract

Leucine zippers are parallel coiled-coil dimers composed of two α-helices with a repeating heptad pattern, designated as *a–g*. Hydrophobic residues at the a and d positions form the dimerization core, whereas charged residues at the *e* and *g* positions generate interhelical electrostatic interactions that influence stability and specificity. Oppositely charged *e–g* pairs are generally considered stabilizing, whereas like-charged pairs are expected to be destabilizing. However, their collective effects across an extended leucine zipper interface have not been systematically evaluated. Here, we designed a 40-residue homodimeric leucine zipper containing 10 *e–g* interaction positions. Ten *e–g* positions were independently assigned to one of three states—neutral, salt-bridge-forming, or repulsive—yielding 59,049 models. Each model was then analyzed with FoldX to calculate the interaction energy and component energy terms. Increasing the number of salt bridges progressively improved the interaction energy, with an average stabilization of 1.68 kcal/mol relative to neutral pairs. Unexpectedly, repulsive pairs were also modestly favorable relative to neutral pairs, improving interaction energy by 0.48 kcal/mol. Energy decomposition showed that favorable solvation and van der Waals contributions outweighed the electrostatic penalty. These effects were position-dependent, indicating that leucine zipper stability reflects a balance of electrostatics, solvation, packing, and positional context.

## 1. Introduction

Proteins perform their biological functions primarily by binding to target molecules, including other proteins. As a result, protein–protein interactions (PPIs) are indispensable to a vast array of cellular processes, ranging from molecular transport and signal transduction to catalysis [1,2,3,4,5,6]. Within these complexes, surface-exposed amino acids dictate the affinity and specificity of the binding interface. Unraveling this specificity is essential for both elucidating fundamental molecular mechanisms and advancing protein engineering. However, deciphering the direct relationship between protein sequence and its interaction remains challenging because of intricate physicochemical factors involved, such as geometric surface complementarity and diverse noncovalent bonds. In this regard, structurally simple model systems serve as invaluable tools for understanding these complex molecular rules.

Among these, interhelical interactions within coiled-coil structures have emerged as a premier model system. This structural motif consists of two or more α-helices twisted around one another to form a superhelical bundle [7]. Although coiled-coils can occur as intramolecular domains within a single protein, they predominantly function as key motifs mediating intermolecular PPIs [8,9,10]. Notable examples of PPI-mediating coiled-coils include basic leucine zipper (bZIP) transcription factors [11], the SNARE complex facilitating membrane fusion [12], and the trimeric assembly domains of TRAF proteins [13].

At the molecular level, the α-helices within a coiled coil interact through hydrophobic residues positioned at the interhelical interface. The hydrophobic side chains of one α-helix pack into the corresponding pockets on the opposite helix—a structural arrangement described by the knobs-into-holes model [14,15]. The amino acid sequences within these interacting regions exhibit a characteristic heptad repeat of seven residues, conventionally designated as positions *a–g* [16]. Among these, positions a and d are typically occupied by hydrophobic residues that form the core hydrophobic interface. The coiled-coil interactions between aligned hydrophobic interfaces alter the geometry of the right-handed α-helix, twisting the helices into a left-handed superhelical structure [17]. Because of their regular, well-defined binding interfaces, coiled-coils serve as ideal model systems for the quantitative analysis of PPI interfaces. Furthermore, they are widely recognized as powerful platforms for engineering synthetic protein assemblies with programmable binding specificity [18,19,20].

Leucine zippers form a dimer through parallel coiled-coil structures characterized by regularly repeated leucine residues at the d position of the heptad repeat. First identified in transcriptional regulatory proteins, they mediate homo- or heterodimerization of DNA-binding transcription factors, such as the bZIP family [14,21]. In addition to hydrophobic interactions, leucine zippers form salt bridges between residues at the *e* and *g* positions, which further stabilize the complex [22]. Although charged residues at *e* and *g* positions are less strictly conserved than the core hydrophobic residues, they are recognized as critical determinants of dimerization specificity. Consequently, electrostatic interactions have become key design elements in engineered coiled-coil systems. Complementary charges promote desired interactions, whereas unwanted interactions are disfavored by removing complementary charged pairs or introducing repulsive pairs [21,23,24]. This rational placement of attractive and repulsive pairs has been widely employed to generate orthogonal coiled-coil pairs for synthetic biology, protein assembly, and molecular recognition applications.

Despite their utility, the precise energetic contributions of electrostatic interactions between the *e* and *g* positions remain incompletely understood. Most previous studies have focused on a small number of mutations or individual salt bridges [25,26], making it difficult to determine how multiple electrostatic interactions act cooperatively across an extended coiled-coil interface. It also remains unclear whether the energetic contribution of an individual *e–g* interaction depends on its position within the heptad repeat, or whether stabilization arises simply from the additive effects of individual salt bridges. Furthermore, although electrostatic interactions are generally interpreted in terms of Coulombic attraction or repulsion, the relative contributions of hydrophobic packing, van der Waals interactions, and solvation energies have not been systematically quantified across a comprehensive sequence landscape.

To address these unresolved questions, we designed a model leucine zipper containing 10 interhelical *e–g* pairs and generated a structural model of the homodimer using AlphaFold3. Using this model as the initial structural template, we utilized FoldX to construct and analyze 59,049 (=310) mutant dimer models, representing every possible combination of neutral, salt-bridge-forming, and ionic-repulsion states at the 10 *e–g* positions. By comprehensively evaluating these variants, we aimed to determine how the number and positional arrangement of electrostatic interactions influence leucine zipper stability and the underlying energetic components. This exhaustive in silico mutagenesis and analysis establishes a quantitative framework for understanding how electrostatic interactions collectively influence leucine zipper stability and provides practical guidelines for the rational engineering of coiled-coil specificity.

## 2. Results

### 2.1. Construction of Mutational Library for Systematic Analysis of e–g Electrostatic Interactions

**Figure 1:**
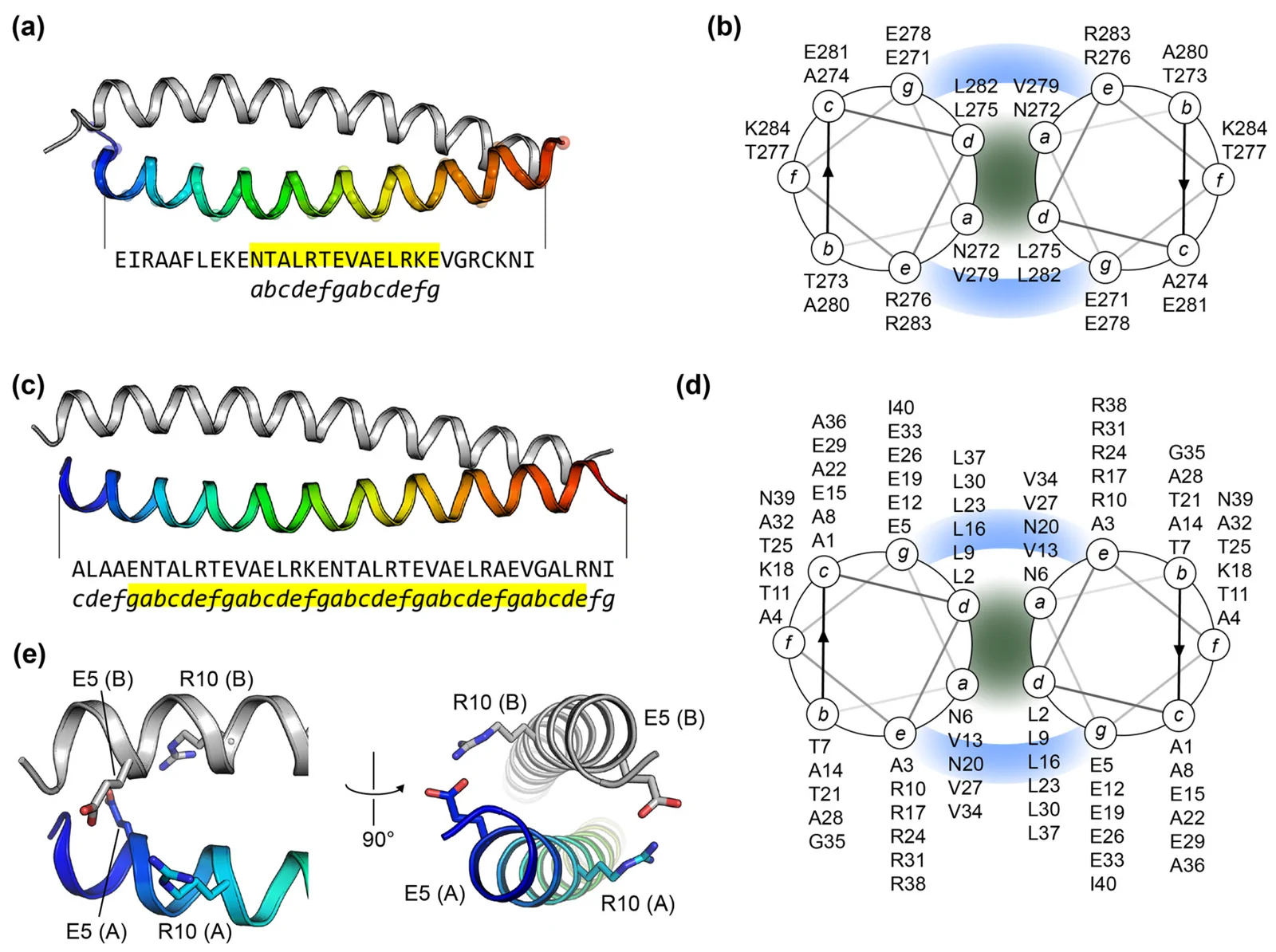
Design and structural modeling of the leucine zipper scaffold for systematic mutational scanning. (**a**) Crystal structure of the leucine zipper region of human thyrotroph embryonic factor (TEF; PDB ID 4U5T), which served as the structural template. One helix is colored progressively from the N-terminus (blue) to the C-terminus (red), and the other helix is shown in gray. The amino acid sequence and residue positions are labeled. The central 14-residue segment selected for duplication and extension is highlighted in yellow. (**b**) Helical-wheel representation of the wild-type TEF leucine zipper homodimer. The heptad-repeat positions are designated by lowercase letters *a–g*. Hydrophobic interactions and salt bridges are shown in green and blue, respectively. (**c**) AlphaFold3-predicted structural model of the designed 40-residue leucine zipper homodimer, generated by extending the central region of the TEF leucine zipper. One helix is colored from the N-terminus (blue) to the C-terminus (red), and the other is shown in gray. The region containing the ten interhelical *e–g* pairs is highlighted in yellow. (**d**) Helical-wheel representation of the designed leucine zipper homodimer. (**e**) Representative structural views of an interhelical Glu–Arg salt bridge within the designed leucine zipper model, shown from side and axial perspectives.

To systematically evaluate how salt bridges and ionic repulsions dictate leucine zipper dimerization, we first constructed the designed homodimeric leucine zipper (LZ-40) scaffold derived from the human thyrotroph embryonic factor (TEF; UniProt ID: Q10587). The crystal structure of TEF (PDB ID: 4U5T) contains a canonical leucine zipper motif, which served as the initial template for engineering an elongated 40-residue helical monomer [27] (Figure 1a,b). The structural model of the LZ-40 homodimer predicted by AlphaFold3 exhibited a canonical parallel coiled-coil conformation with high structural confidence (ipTM exceeding 0.81) (Figure 1c). Structural analysis of this homodimer model successfully mapped 10 interhelical *e–g* interaction sites: E5(g)–R10(e), R10(e)–E5(g), E12(g)–R17(e), R17(e)–E12(g), E19(g)–R24(e), R24(e)–E19(g), E26(g)–R31(e), R31(e)–E26(g), E33(g)–R38(e), and R38(e)–E33(g) (Figure 1d). The side chains at these *e–g* pairs were within a distance range of 2.5–3.1 Å, confirming their geometric competence for robust salt-bridge formation (Figure 1e).

**Figure 2:**
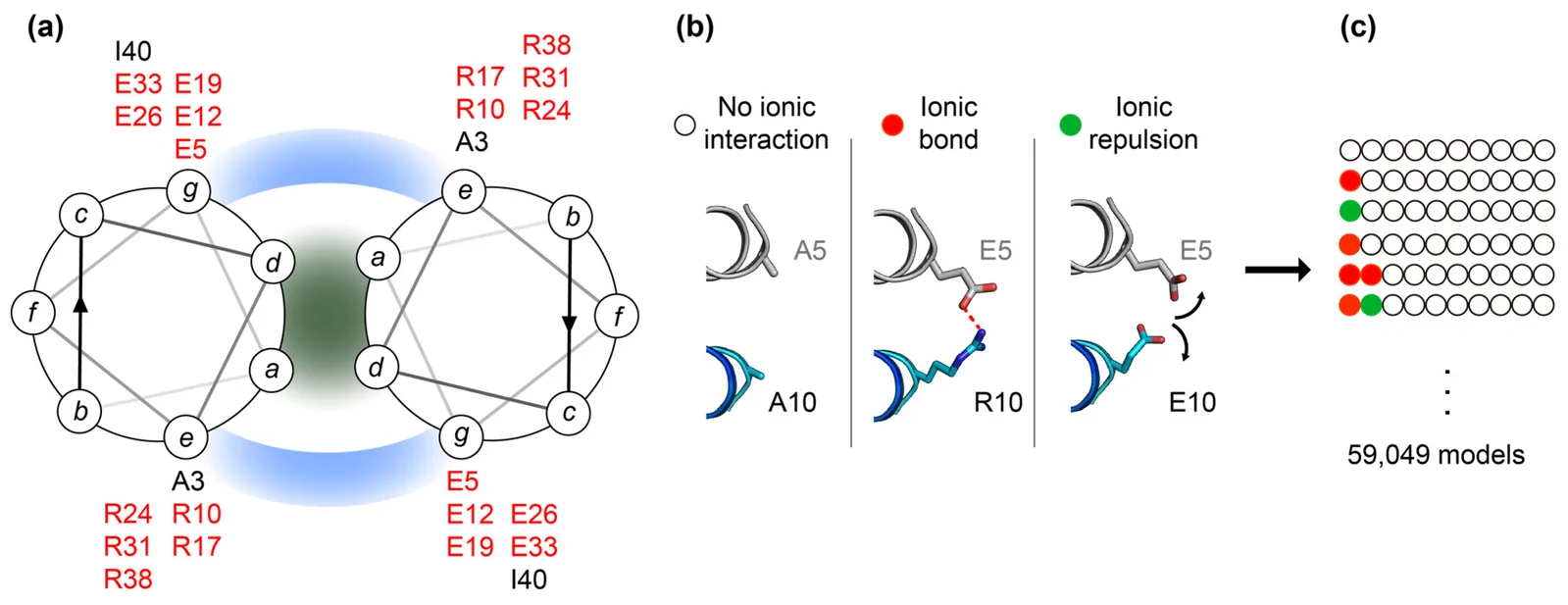
Design of the systematic mutational library for interhelical *e–g* interactions. (**a**) Helical-wheel representation of the designed leucine zipper homodimer. The 10 interhelical *e–g* pairs selected for mutational scanning are red. (**b**) Structural representation of the three interaction states assigned to each *e–g* pair: neutral Ala–Ala pair (N; white), salt-bridge-forming Glu–Arg or Arg–Glu pair (S; red), and like-charged Glu–Glu or Arg–Arg repulsive pair (R; green). Representative side-chain conformations are shown for each state. (**c**) Schematic representation of the combinatorial sequence library. Each row represents a dimer variant, and each circle represents one of the 10 *e–g* pair positions. White, red, and green circles indicate neutral, salt-bridge-forming, and repulsive states, respectively. Independent assignment of the three states at all 10 positions generated 59,049 (=3^10^) dimer variants.

Ten *e–g* pairs were systematically mutated to evaluate all possible combinations of three electrostatic interaction states: attractive, neutral, and repulsive (Figure 2a). For the attractive state, the *e–g* pair was left unmutated as either an E(e)–R(g) or R(g)–E(e) pair and was classified as a salt-bridge-forming pair (S). For the neutral state, both residues of the *e–g* pair were simultaneously mutated to alanine, yielding an A(e)–A(g) pair classified as a neutral pair (N). For the repulsive state, the residue in Chain B was mutated to match the corresponding charged residue in Chain A, generating either an E(e)–E(g) or R(e)–R(g) pair, which was classified as a repulsive pair (R). These mutations were independently introduced at each of the 10 *e–g* pairs, generating 59,049 (310) models, including the template model with 10 salt-bridge-forming pairs (Figure 2b). For each mutant dimer, the FoldX interaction energy and its component energy terms—including electrostatic, solvation, and van der Waals energies—were evaluated. Across the 59,049 models, the mean interaction energy was −40.69 ± 2.59 kcal/mol. The original model containing 10 salt bridges exhibited the lowest (most favorable) interaction energy of −51.01 kcal/mol. In contrast, the model with three repulsive and seven neutral *e–g* pairs (NRNNNRRNNN) had the highest (least favorable) binding free energy of −33.74 kcal/mol.

### 2.2. Global Energetic Effects of Salt Bridges and Ionic Repulsions

**Figure 3:**
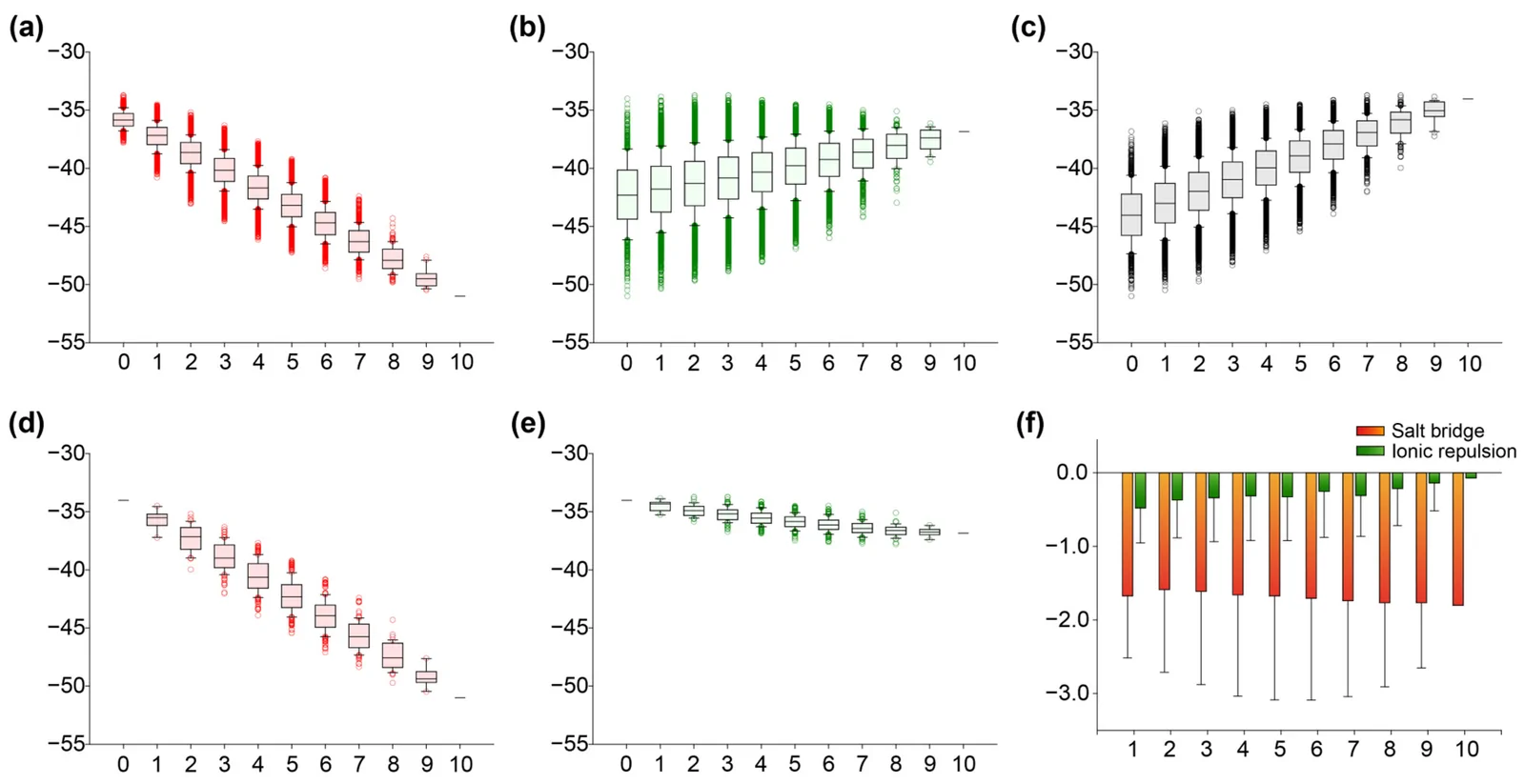
Global effects of salt bridges, repulsive pairs, and neutral pairs on FoldX interaction energy. (**a**–**c**) Boxplots of FoldX interaction energy for the complete library of 59,049 dimer variants, grouped by the number of (**a**) salt bridges, (**b**) repulsive pairs, or (**c**) neutral Ala–Ala pairs. The x-axis indicates the number of the corresponding pair type, and the y-axis indicates FoldX interaction energy (kcal/mol). (**d**) Interaction energies of the 1024 variants containing exclusively salt-bridge-forming and neutral pairs, plotted as a function of the number of salt bridges. (**e**) Interaction energies of the 1024 variants containing exclusively repulsive and neutral pairs, plotted as a function of the number of repulsive pairs. For all boxplots, boxes represent the interquartile range, center lines indicate the median, whiskers extend to 1.5 × the interquartile range, and individual points indicate outliers. (**f**) Mean change in interaction energy associated with the sequential addition of each salt bridge or repulsive pair. Red and green bars indicate the effects of adding salt-bridge-forming and repulsive pairs, respectively. Negative values indicate more favorable interaction energies.

To assess the energetic impacts of salt bridges and ionic repulsions at the *e–g* pairs on the leucine zipper interface, the FoldX interaction energies of the 59,049 models were compared as a function of the number of salt bridges, repulsive pairs, and neutral pairs (Figure 3a–c). As the number of salt bridges increased, the interaction energy became progressively more negative, decreasing the free energy from −35.82 to −51.01 kcal/mol, with a strong negative correlation (R2 = 0.735). A linear regression describing this trend yielded a slope of −1.489 kcal/mol per additional salt bridge (Figure 3c). In contrast, increasing the number of ionic repulsions shifted the interaction energy toward less negative values, rising from −42.27 to −36.84 kcal/mol, although the correlation was weak (R2 = 0.082). Similarly, increasing the number of neutral pairs shifted the interaction energy toward less negative values, from −44.01 to −34.03 kcal/mol with a modest correlation (R2 = 0.327) (Figure 3b,c). These results indicate that salt bridges strongly stabilize the binding interface, whereas the destabilizing effects observed upon accumulating neutral or repulsive pairs in the full library were weaker and highly dependent on the specific combinations of states at the remaining *e–g* positions (Figure 3b,c).

Because the numbers of neutral pairs, salt bridges, and ionic repulsions strictly sum to 10, increasing the number of one state inherently constrains and decreases the numbers of the other two states. To circumvent this compositional constraint imposed by the fixed number of *e–g* positions, restricted model subsets were defined and analyzed. First, to isolate the effect of salt bridges from that of ionic repulsions, interaction energies were calculated for a subset of 1024 models containing exclusively neutral and salt-bridge pairs, with no repulsive pairs. As salt bridges accumulated across the 10 *e–g* positions, the interaction energy became progressively more negative, decreasing from −34.03 to −51.01 kcal/mol with a strong negative correlation (R^2^ = 0.735). Linear regression yielded a slope of −1.69 kcal/mol per additional salt bridge (Figure 3d). This robust and consistent relationship demonstrates that the stabilizing contribution of salt bridges is cumulative and remains evident even in the absence of repulsive pairs. Second, to isolate the effect of ionic repulsions, a separate subset of 1024 models containing only neutral and repulsive pairs (no salt bridges) was analyzed. Counterintuitively, increasing the number of repulsive pairs across the 10 *e–g* positions produced a slight but progressive decrease in interaction energy, dropping from −34.03 to −36.84 kcal/mol with a weak negative correlation (R^2^ = 0.384), with a slope of −0.29 kcal/mol per additional repulsive pair (Figure 3e).

The incremental changes in interaction energy resulting from each additional salt-bridge or ionic repulsion were further compared. Each additional salt bridge resulted in a favorable change in interaction energy ranging from −1.59 to −1.80 kcal/mol, and the magnitude of this stabilizing effect tended to increase slightly as salt bridges accumulated. However, the addition of an ionic-repulsion pair also yielded a modest, favorable energy change in Interaction Energy, ranging from −0.07 to −0.48 kcal/mol (Figure 3f).

The effect of ionic repulsions differed between the full-library analysis and the subset without salt bridges (Figure 3b,e). In the full library, an increase in the number of ionic repulsions implicitly replaced either a stabilizing salt-bridge or a neutral pair. Consequently, the effect of ionic repulsion was confounded by variation in the number of salt bridges, resulting in less favorable average interaction energies. In contrast, within the subset devoid of a salt bridges, replacing a neutral pair with an ionic-repulsion pair produced a small but favorable change in interaction energy. This finding is contrary to the conventional view that identical charged residues at *e–g* pairs destabilize the binding interface through electrostatic repulsion [25,28].

### 2.3. Energetic Basis of Salt-Bridge and Ionic-Repulsion Effects

**Figure 4:**
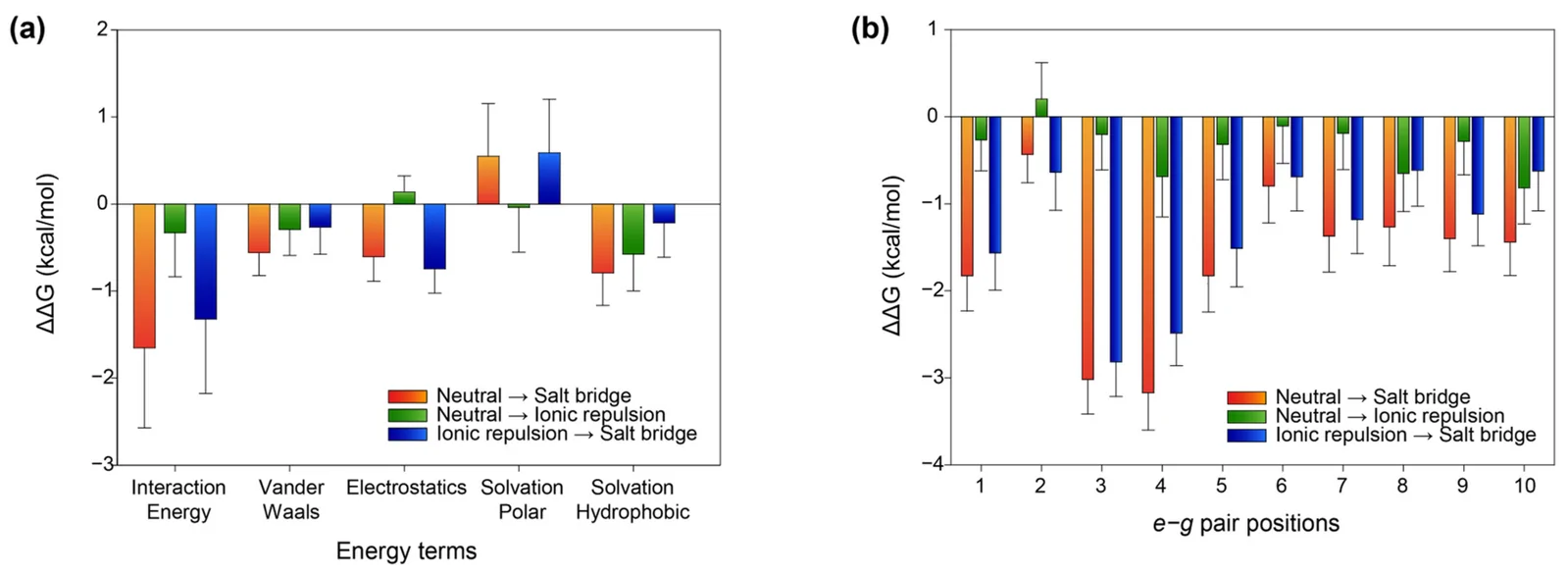
Energy decomposition of salt-bridge-forming and repulsive *e–g* pairs. (**a**) Mean changes in FoldX interaction energy and component energy terms associated with replacing a neutral Ala–Ala pair with either a salt-bridge-forming pair or a like-charged repulsive pair. The analyzed terms include interaction energy, electrostatics, van der Waals, solvation hydrophobic, and solvation polar. Red, green, and blue bars represent the neutral-to-salt-bridge, neutral-to-repulsive, and repulsive-to-salt-bridge comparisons, respectively. Negative values indicate favorable energetic contributions, whereas positive values indicate unfavorable contributions. (**b**) Position-dependent decomposition of the energetic effects at each of the 10 interhelical *e–g* pair positions. For each position, bars show the changes in interaction energy and the major component energy terms for the neutral-to-salt-bridge, neutral-to-repulsive, and repulsive-to-salt-bridge comparisons. Negative values indicate stabilization relative to the corresponding reference state.

The energetic effects of salt bridges and ionic repulsions were further evaluated by directly comparing each state with the alanine baseline. Models were grouped into triplets differing only in the mutational state of a single *e–g* pair, whereas all other pairs remained identical. For example, NNNNNNNNNN, SNNNNNNNNN, and RNNNNNNNNN formed one triplet, where N, S, and R represent the neutral, salt-bridge-forming, and repulsive states, respectively. As expected, replacing a neutral state with a salt-bridge-forming state stabilized the interface, yielding a favorable change in FoldX interaction energy (ΔΔGint) of −1.65 ± 0.92 kcal/mol on average. Surprisingly, replacing the neutral state with a repulsive state also stabilized the dimer, yielding a mean change in interaction energy of −0.33 ± 0.507 kcal/mol (Figure 4a). To assess the energetic basis of these effects, we analyzed four constituent FoldX energy terms: electrostatics (electrostatic interactions), van der Waals (van der Waals interactions), solvation polar (penalization for burying polar groups), and solvation hydrophobic (contribution of hydrophobic groups). For salt bridges, the electrostatics, van der Waals, and solvation hydrophobic terms contributed favorably, whereas the solvation polar term contributed unfavorably (Figure 4a). The introduction of oppositely charged side chains produced a strong Coulombic attraction, resulting in a favorable electrostatic contribution of −0.56 kcal/mol upon binding. Because the side chains of glutamate (Glu) and arginine (Arg) contain extended aliphatic carbon chains, bringing them into proximity enhanced nonpolar packing, which contributed favorable changes of −0.82 kcal/mol to the solvation hydrophobic term and −0.54 kcal/mol to the van der Waals term. Conversely, pairing these oppositely charged groups required the partial desolvation of their ionic moieties, resulting in an unfavorable solvation polar penalty of +0.46 kcal/mol.

For ionic-repulsion pairs, only the electrostatics term contributed unfavorably, whereas the van der Waals, solvation hydrophobic, and solvation polar terms all made favorable contributions (Figure 4a). The introduction of same-charged residues produced Coulombic repulsion, imposing an electrostatic penalty of +0.11 kcal/mol. This penalty represents the direct electrostatic cost of positioning an ionic repulsion at an *e–g* pair. The van der Waals term made a moderately favorable contribution of −0.27 kcal/mol, likely because replacing alanine with bulkier charged residues increased the side-chain surface area available for packing contacts with neighboring residues. However, unlike in a salt bridge, electrostatic repulsion between identical charges pushes the two side chains apart, preventing them from achieving near-optimal packing distance. Consequently, the favorable van der Waals contribution was less pronounced than that of the salt-bridge-forming state. The solvation hydrophobic term also contributed favorably by −0.62 kcal/mol. This suggests that the aliphatic portions of the charged residues may still contribute to the burial of nonpolar surface area through hydrophobic packing with adjacent hydrophobic residues at the interface, even as the charged termini repel each other. Notably, the solvation polar term was also favorable, contributing −0.15 kcal/mol on average. Because same-charged residues repel each other, they possibly adopt more extended, solvent-exposed conformations. This allows the charged headgroups to remain highly solvated by bulk water, effectively minimizing the desolvation penalty that typically accompanies salt-bridge formation. Although the electrostatics term was unfavorable, the cooperative favorable contributions of the other three energy terms outweighed this penalty, yielding an overall net negative stabilization energy. These findings demonstrate that harboring an ionic repulsion at an *e–g* position is energetically far less detrimental than would be anticipated based solely on classical Coulombic repulsion.

### 2.4. Position-Dependent Effects of Salt Bridges and Ionic Repulsions

To further characterize the spatial effects of salt bridges and ionic repulsions along the extended coiled-coil interface, we analyzed their constituent energy terms across the 10 individual *e–g* pair positions. A single salt bridge exerted the most pronounced stabilizing effects at the third and fourth *e–g* positions, yielding mean changes in FoldX interaction energy of −3.02 and −3.17 kcal/mol, respectively. At the remaining positions, each salt bridge produced smaller favorable contributions, with mean changes in interaction energy ranging from −0.79 to −1.83 kcal/mol, whereas the salt bridge at the second *e–g* position exhibited the weakest stabilization at −0.43 kcal/mol (Figure 4b).

The enhanced stabilization observed at some *e–g* positions may reflect differences in the local structural environment, including side-chain geometry, packing, solvent exposure, and conformational flexibility. However, these structural features were not directly quantified in the present analysis and therefore should be regarded as possible explanations rather than direct mechanistic conclusions. Additional backbone-sensitivity analyses showed that the pronounced stabilization observed at several salt-bridge positions was not consistently reproduced in two independently generated starting backbone models. The positional salt-bridge profiles showed low correlations with the original model (Pearson r = 0.23 and −0.14), indicating that the precise magnitude and positional ranking of salt-bridge stabilization are sensitive to the local backbone geometry. Nevertheless, the overall tendency for salt-bridge substitutions to provide favorable energetic contributions was retained, suggesting that the backbone dependence primarily affects the quantitative magnitude and relative ranking of individual positions rather than the general stabilizing effect of salt-bridge formation.

In contrast, ionic repulsions exhibited a less predictable, position-dependent energetic profile. Their thermodynamic contributions followed a non-monotonic, zigzag pattern, with mean changes in interaction energy spanning from +0.20 to −0.82 kcal/mol. The relative positional profiles of repulsive substitutions were more reproducible across the alternative backbone models than those of salt bridges (Pearson r = 0.77–0.79 relative to the original model). Additional side-chain rebuilding tests also reproduced the overall repulsive-pair profile well when the variants were rebuilt from a common neutral template (Pearson r = 0.93). However, reversing the order in which the two interfacial mutations were introduced produced appreciable energy differences at several positions, particularly positions 2, 6, and 8. Thus, although the overall context-dependent energetic behavior of the repulsive substitutions was retained, the exact magnitude of the effect at particular positions was sensitive to local side-chain optimization and the rebuilding pathway.

The energetic difference between the salt-bridge-forming and ionic-repulsion states revealed pronounced site-dependent effects. At all 10 *e–g* pair positions, the salt-bridge-forming state was more favorable than the corresponding repulsive state, with the mean energetic advantage (interaction energySB—interaction energyRP) ranging from −2.82 to −0.62 kcal/mol. The largest energy differences were observed at the third and fourth positions, displaying values of −2.82 and −2.49 kcal/mol, respectively. Intermediate differences were observed at the first, fifth, seventh, and ninth positions (ranging from −1.56 to −1.12 kcal/mol), whereas the gaps narrowed considerably at the second, sixth, eighth, and tenth positions, where values ranged from −0.61 to −0.69 kcal/mol (Figure 4b). Together, these results demonstrate that the energetic advantage of a salt-bridge-forming state over a repulsive state varies along the coiled-coil interface, although the precise magnitude and positional ranking of these effects are influenced by the local structural environment and the side-chain rebuilding procedure.

### 2.5. Non-Additive Energetic Coupling Between Neighboring e–g Interactions

**Figure 5:**
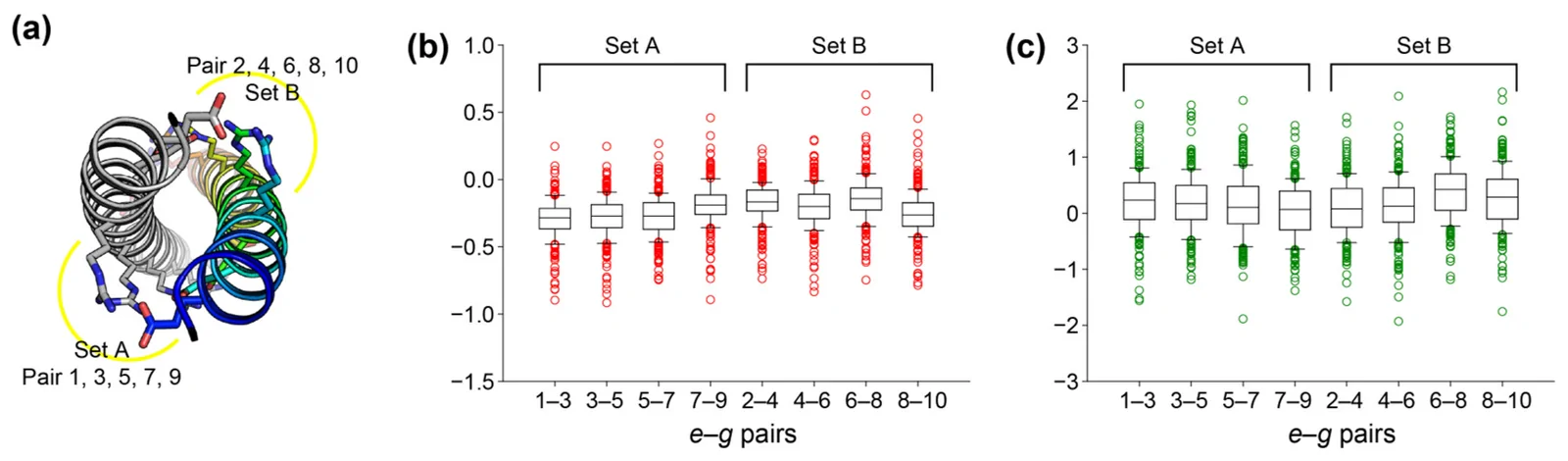
Energetic coupling between two adjacent *e–g* pairs. (**a**) Two sets of *e–g* pairs aligned along the coiled-coil interface are shown as stick diagram. Distribution of coupling energies for (**b**) adjacent salt-bridge-forming pairs in the 1024 variants without repulsive pairs, and (**c**) adjacent repulsive pairs in the 1024 variants without salt-bridge-forming pairs are shown as box plots. For all boxplots, boxes represent the interquartile range, center lines indicate the median, whiskers extend to 1.5 × the interquartile range, and individual points indicate outliers.

To determine whether multiple *e–g* interactions contribute independently or exhibit energetic coupling, we calculated a coupling energy from two adjacent *e–g* pairs. The 1024 variants containing exclusively salt-bridge-forming and neutral pairs, and the 1024 variants containing exclusively repulsive and neutral pairs were separately prepared. In each subset, the 10 *e–g* pair positions were divided into two sets according to their alignment along the coiled-coil interface: Set A (1, 3, 5, 7, and 9 *e–g* pair positions) and Set B (2, 4, 6, 8, and 10 *e–g* pair positions). For each pair of positions within a set, coupling energy was calculated as the deviation in the double-substitution effect from the sum of the two corresponding single-substitution effects, using the neutral state as the reference across all 256 (=2^8^) possible sequence backgrounds (Figure 5a). Values near zero indicate approximately additive behavior, whereas negative values indicate that the combined substitutions are more favorable than expected from additivity, and positive values indicate that they are less favorable.

Adjacent salt bridges exhibited negative mean coupling energies, ranging from −0.30 to −0.14 kcal/mol across the eight neighboring position pairs (Figure 5b). The mean coupling energies for adjacent pairs were −0.238 and −0.215 kcal/mol for Sets A and B, respectively, whereas those for more distant pairs were close to zero (+0.001 and −0.010 kcal/mol, respectively). These results indicate that neighboring salt bridges tend to exhibit cooperative stabilization, whereas the energetic contributions of more spatially separated salt bridges are largely additive. Nevertheless, the distributions of coupling energies were broad across the 256 sequence backgrounds, indicating that the magnitude of this cooperative effect depends substantially on the surrounding electrostatic context.

In contrast, neighboring repulsive pairs exhibited positive mean coupling energies, ranging from +0.04 to +0.39 kcal/mol (Figure 5c). The mean coupling energies of adjacent repulsive pairs were +0.151 and +0.213 kcal/mol for Sets A and B, respectively, compared with values close to zero for more distant pairs (+0.012 and +0.003 kcal/mol). Positive coupling indicates that the combined effect of two neighboring repulsive substitutions is less favorable than expected from the sum of their individual effects, consistent with anti-cooperative energetic coupling. However, coupling energies are distributed broadly across both positive and negative values, again demonstrating a strong dependence on the surrounding electrostatic context.

## 3. Discussion

Our study demonstrates that leucine zipper stability is governed by a balance of multiple energetic contributions rather than by electrostatics alone. Although same-charged *e–g* pairs would be expected to destabilize coiled-coil interactions from a purely charge-based perspective, they were more favorable than the Ala–Ala baseline in our model. Energy decomposition indicated that favorable contributions from solvation, hydrophobic, and van der Waals terms outweighed the unfavorable electrostatics contribution. Thus, relative to alanine, glutamate and arginine residues contribute not only charged groups but also substantial aliphatic surface areas that participate in hydrophobic burial and side-chain packing contacts. The larger side-chain volumes of Glu and Arg relative to Ala may therefore contribute to the observed favorable net energies. Accordingly, these results should be interpreted as the net energetic consequence of introducing like-charged pairs rather than as evidence that electrostatic repulsion itself is stabilizing. A more rigorous distinction between charge effects and side-chain-size effects would require additional size-matched neutral controls.

A key energetic distinction between salt-bridge-forming and repulsive pairs was observed in the solvation polar term. Salt-bridge formation brought oppositely charged groups into proximity and incurred an unfavorable desolvation contribution. In repulsive pairs, by contrast, like-charged side chains may adopt more extended and solvent-exposed conformations, allowing their polar and charged moieties to remain favorably solvated. This thermodynamic difference effectively narrowed the energetic gap between salt-bridge-forming and repulsive pairs relative to what would be anticipated from electrostatics alone. Because side-chain orientation and solvent exposure were not directly quantified in the present analysis, however, these structural interpretations should be regarded as possible explanations rather than direct mechanistic conclusions.

Despite being energetically more favorable than the Ala–Ala baseline, repulsive pairs remained less favorable than salt bridges at all 10 *e–g* positions. Replacing a salt bridge with a repulsive pair therefore weakened the interhelical interaction, although the magnitude of this destabilization varied substantially across the coiled-coil interface. These findings indicate that the energetic consequences of charged-residue substitutions depend strongly on their positional context. Additional rebuilding analyses further showed that the magnitude and positional ranking of these effects, particularly those of salt bridges, can depend on the starting backbone structure and side-chain reconstruction pathway. Thus, the precise energetic contribution assigned to an individual *e–g* position should be interpreted as context-dependent rather than as an intrinsic property of that position. In addition, analysis of neighboring *e–g* pairs revealed non-additive energetic coupling. Adjacent salt bridges tended to exhibit negative coupling energies, consistent with cooperative stabilization, whereas adjacent repulsive pairs generally showed positive coupling energies, indicating that their combined effects were less favorable than expected from simple additivity. The broad distributions of coupling energies across different sequence backgrounds further indicate that the magnitude of these effects is strongly modulated by the surrounding interaction network.

These findings have important implications for the interpretation and rational design of leucine zipper interactions. Rather than being determined by electrostatics alone, the energetic consequences of individual *e–g* pairs reflect the combined effects of charge complementarity, local side-chain geometry, packing, solvation, and energetic coupling with neighboring interactions. In particular, a single repulsive *e–g* pair may not provide a sufficiently robust specificity determinant, because the magnitude of its energetic effect can depend strongly on the local structural context, whereas combinations of neighboring electrostatic interactions may exhibit non-additive coupling. Although the present study employed a simplified synthetic leucine-zipper model, these observations may provide useful qualitative guidance for natural coiled-coil systems. When natural leucine zippers are analyzed or engineered, *e–g* charge patterns should therefore be considered together with solvent exposure, hydrophobic core packing, neighboring electrostatic networks, energetic coupling between adjacent interactions, and the relative alignment of the helices. Accordingly, the quantitative energetic values obtained here should not be regarded as universal rules for individual positions, but rather as context-dependent design principles that can help prioritize candidate interactions or mutations for subsequent structure-specific computational and experimental evaluation.

## 4. Materials and Methods

### 4.1. Structural Model Preparation

Residues 258–294 of chain A in the crystal structure of TEF (PDB ID: 4U5T) [27] were used as the initial template to design an extended leucine zipper. To construct this elongated scaffold, the central 14-residue segment (residues 271–284) was duplicated twice, and the N- and C-terminal residues were modified to eliminate potential charged interactions and disulfide bonds. This engineering process yielded a de novo 40-residue sequence designated as LZ-40: ALAAENTALRTEVAELRKENTALRTEVAELRAEVGALRNI. The structural model of the LZ-40 homodimer was subsequently predicted using AlphaFold3 (London, United Kingdom) [29]. All five output models were predicted with high confidence, exhibiting ptm and iptm values greater than 0.81. The model with the highest-ranking score was selected for subsequent computational analysis.

### 4.2. Mutational Sequence Library Preparation

The LZ-40 homodimer model was manually inspected using PyMOL 2.6.0a0 (New York, United States) [30], and the repeated leucine residues were assigned to the d positions of the heptad repeat. Based on this topological assignment, 10 *e–g* pairs were identified, each capable of forming an interhelical salt bridge between position *e* of Chain A and position *g* of Chain B, or vice versa (position *g* of Chain A and position *e* of Chain B). Each *e–g* pair was independently assigned to one of three mutational states: neutral (N), salt-bridge-forming (S), or repulsive (R), yielding a combinatorial library of 59,049 (310) variants. For a neutral pair, both amino acids were mutated to alanine. For a salt-bridge-forming pair, the wild-type residues were retained without mutation. For a repulsive pair, the amino acid in Chain B was mutated to match the corresponding residue in Chain A. A total of 59,048 mutant sequences were generated using a custom Python script and formatted line-by-line within a single input file for high-throughput FoldX calculations.

### 4.3. Mutant Model Generation and FoldX Interaction Energy Analysis

The structural model generated by AlphaFold3 was prepared for analysis with FoldX 5 using the RepairPDB command of FoldX 5 (Barcelona, Spain) [31]. The repaired model and the mutational library were then used as inputs for the BuildModel, which was run once for each mutant. Each mutant dimer model was subsequently analyzed using the AnalyseComplex command in FoldX 5. The RepairPDB, BuildModel, and AnalyseComplex commands were executed under default settings.

The Interaction Energy reported by AnalyseComplex was used as the FoldX estimate of the energetic favorability of the interaction between the two helices. More negative Interaction Energy values were interpreted as indicating more favorable interhelical interactions. To evaluate the energetic change associated with each additional salt bridge or repulsive pair, the change in FoldX Interaction Energy was calculated as

$${∆IE}^{Si}=\bar{{IE}^{Si}}-\bar{{IE}^{S(i-1)}}$$

$${∆IE}^{Ri}=\bar{{IE}^{Ri}}-\bar{{IE}^{R(i-1)}}$$

where $\bar{{IE}^{Si}}$ and $\bar{{IE}^{Ri}}$ denote the mean FoldX interaction energies of models containing *i* salt bridges and *i* ionic repulsions, respectively. Negative ∆IE values indicate that adding an interaction is associated with a more favorable interaction energy.

### 4.4. Pairwise Comparison of e–g Interaction States

For position-specific pairwise comparisons, models with identical states at nine *e–g* positions but differing at the remaining comparison position were grouped into triplets. Each triplet contained a neutral state (N), a salt-bridge-forming state (S), and an ionic-repulsion state (R) at the comparison position. This procedure generated 196,830 (39 × 10) triplets. Within each triplet, differences in interaction energy were calculated as follows:

$$∆IE_{N\to S}=IE_{S}-IE_{N}$$

$$∆IE_{N\to R}=IE_{R}-IE_{N}$$

$$∆IE_{R\to S}=IE_{S}-IE_{R}$$

where $IE_{N}$, $IE_{S}$, and $IE_{R}$ denote the interaction energy values of the model containing a neutral pair, a salt-bridge-forming pair, and an ionic-repulsion pair, respectively, at the comparison site of a triplet. Negative values indicate that the state listed after the arrow has a more favorable interaction energy than the reference state listed before the arrow.

### 4.5. Data Processing, Statistical Analysis, and Visualization

Mutation lists and FoldX output tables were prepared and summarized using custom Python 3.11 scripts (Python Software Foundation, Beaverton, OR, USA). Data handling, summary statistics, and figure generation were performed using the pandas, numpy, and matplotlib libraries. Sample size (n), mean, and standard deviation (SD) were explicitly calculated for grouped comparisons. Mean values are presented as mean ± SD unless otherwise indicated. Structural figures were rendered in PyMOL [30], and statistical graphics were generated using SigmaPlot. During the preparation of this manuscript, the authors used ChatGPT 5.4 (OpenAI) to assist in drafting custom Python scripts for data processing and analysis and in improving the clarity and readability of the text. Following the use of this tool, the authors thoroughly reviewed, edited, and validated all code and text, and they take full responsibility for the final content of the publication.

## Data Availability

The data supporting the findings of this study, including structural models, FoldX analysis outputs, and analysis scripts, are available on Zenodo (DOI: 10.5281/zenodo.22137120).

## Abbreviations

The following abbreviations are used in this manuscript:

PPI	Protein–Protein Interaction
bZIP	Basic Leucine Zipper
TEF	Thyrotroph Embryonic Factor
LZ-40	40-residue Designed Leucine Zipper
ipTM	Interface Predicted Template Modeling score
pTM	Predicted Template Modeling score
SD	Standard Deviation
Glu	Glutamate
Arg	Arginine
Ala	Alanine
